# Anticancer Effects of *Weizmannia coagulans* MZY531 Postbiotics in CT26 Colorectal Tumor-Bearing Mice by Regulating Apoptosis and Autophagy

**DOI:** 10.3390/life14101334

**Published:** 2024-10-19

**Authors:** Bao Zhong, Yujuan Zhao, Lei Gao, Ge Yang, Yansong Gao, Fenglin Li, Shengyu Li

**Affiliations:** 1 Institute of Agro-Food Technology, Jilin Academy of Agricultural Sciences (Northeast Agricultural Research Center of China), Changchun 130033, China; zhongbao19870626@163.com (B.Z.); sunny@126.com (Y.Z.); redhuman@126.com (L.G.); yangge1900@163.com (G.Y.); gysgerry@126.com (Y.G.); 2 College of Food Science and Nutritional Engineering, Jilin Agriculture Science and Technology University, Jilin 132101, China; swgclfl@163.com

**Keywords:** *Weizmannia coagulans*, postbiotics, colorectal cancer, apoptosis, autophagy

## Abstract

*Weizmannia coagulans* has been shown to have anticancer properties. However, there is limited research on the effects of postbiotic *W. coagulans* on colorectal cancer cell proliferation. Additionally, the exact mechanisms through which it influences apoptosis- and autophagy-related signaling pathways are yet to be thoroughly elucidated. This study explored the role of *W. coagulans* MZY531 as a postbiotic in inhibiting tumor growth by modulating apoptosis and autophagy in tumor cells. During the experimental period in the model group, tumors proliferated, tumor markers increased significantly, and immunofluorescence results showed that caspase-3 and terminal deoxynucleotidyl transferase dUTP nick-end labeling were significantly decreased. Conversely, supplementation with *W. coagulans* MZY531 postbiotics significantly reduced the levels of tumor markers carcinoembryonic antigen, colon cancer antigen, and extracellular protein kinase A and promoted cell apoptosis by increasing the caspase-3-positive count and terminal deoxynucleotidyl transferase dUTP nick-end labeling-positive cells in tumor tissue. Mechanistically, *W. coagulans* MZY531 postbiotics inhibit tumor growth through the modulation of the Bax/Bcl-2/caspase-3 and JAK2/STAT3 apoptosis pathways and PI3K/AKT/mTOR and TGF-β/SMAD4 cell autophagy pathways. *W. coagulans* MZY531 postbiotics had a more significant effect than that of *W. coagulans* MZY531 alone. Probiotics are expected to become effective natural functional foods for the treatment of colorectal cancer.

## 1. Introduction

In 2020, colorectal cancer (CRC) accounted for 10% of global cancer cases and 9.4% of cancer-related fatalities, making it the second leading cause of cancer-related mortality after lung cancer, responsible for 18% of such deaths. The global incidence of CRC is projected to reach 3.2 million new cases by 2040, which is influenced by factors such as aging, population growth, and advances in human development [1,2]. Increased exposure to environmental risk factors is the main reason for the increase in CRC incidence and is linked to lifestyle and dietary changes associated with Westernization [3]. The treatments for CRC include surgery, chemotherapy, radiotherapy, and targeted therapies. However, targeted therapies are still at an exploratory stage [4]. Surgery and adjuvant chemotherapy are associated with considerable adverse effects [5]. Currently, the prevention and treatment of CRC using natural substances or traditional medicines is an important direction for researchers to explore [6,7]. The onset of CRC is associated with a decrease or increase in specific bacterial species in the gut microbiome of patients [8]. Therefore, anti-CRC therapy based on the intestinal flora has become a new research direction.

Probiotics, as beneficial intestinal bacteria, have shown advantages in preventing CRC and exerting anti-proliferative effects by regulating cell apoptosis and differentiation [9]. Research suggests that probiotics may inhibit the proliferation of the bacteria responsible for converting procarcinogens into active carcinogens. This action reduces the overall carcinogen load in the intestine, thereby lowering the risk of CRC [10]. Potential mechanisms of action of probiotics in preventing CRC include changing the intestinal flora, improving the physical and chemical conditions of the colon, producing anticancer and antioxidant metabolites, reducing intestinal inflammation, inhibiting harmful enzymes, and promoting apoptosis [11,12]. Bioactive components produced by viable probiotics or released following bacterial lysis are defined as postbiotics [13]. Postbiotics are novel biological substances that do not contain living microorganisms. Research on postbiotics is still in its early stages; however, increasing evidence suggests that postbiotics offer significant advantages in terms of immunomodulation, anti-inflammatory effects, and anticancer properties [14]. Postbiotic supplementation may enhance CRC management through mechanisms such as immune system activation, suppression of cancer cell growth, induction of apoptosis and necrosis in tumor cells, and modulation of the gut microbiome in affected individuals [15].

*Weizmannia coagulans* is a spore-forming probiotic that is currently utilized extensively in medicine, food, and other fields. *W. coagulans* has therapeutic potential for a range of intestinal disorders by modulating the host gut microbiota [16,17]. *W. coagulans* exhibits antimicrobial activity against pathogenic intestinal organisms in patients with colitis [18]. Heat-killed *B. coagulans* culture supernatant effectively induces apoptosis in colon cancer cells by increasing the expression of the proapoptotic proteins Bax and caspase-3 and decreasing the expression of the antiapoptotic protein Bcl-2 [19]. In addition, *W. coagulans* MZY531 is advantageous in regulating breast and liver cancers [20,21]. The anti-CRC mechanism of *W. coagulans* MZY531 requires further exploration. Research on postbiotics is more interesting than that on probiotics. Therefore, the aim of this study was to investigate the potential mechanism by which *W. coagulans* MZY531 alleviates CRC by regulating autophagy and apoptosis.

## 2. Materials and Methods

### 2.1. Weizmannia coagulans MZY531 Culture Methods and Postbiotics Preparation

The strain of *W. coagulans* MZY531 ( Jilin Province Mingzhiyuan Biotechnology Co., Ltd., Changchun, China) was activated on a Luria–Bertani (LB) solid plate, inoculated into glucose yeast extract peptone (GPY)liquid broth, and shaken (1800 rpm) for 24 h at 50 °C. After centrifugation, the bacterial precipitate was collected (3000 rpm, 4 °C, 10 min) and then suspended in a sterile isotonic sodium chloride solution, adjusting the concentration of the bacterial solution to 1.0 × 10^9^ CFU/mL. Fifty milliliters of *W. coagulans* MZY531 suspension (1.0 × 10^9^ CFU/mL) was subjected to ultrasonic disruption in an ice bath for 15 min at 800 W using an ultrasonic disruptor. The obtained suspension was processed by vacuum freeze drying to produce a concentrated powder of *W. coagulans* MZY531 postbiotic. This powder was dissolved in 50 mL of physiological saline to create an oligosaccharide suspension for use in subsequent animal studies.

### 2.2. Cell Line and Culture Condition

The mouse colon carcinoma CT26.WT cells were purchased from Jiangsu KeyGEN BioTech Co., Ltd. (Nanjing, China). These cells were cultured in RPMI-1640 medium supplemented with 10% fetal bovine serum, 100 IU/mL penicillin, and 100 µg/mL streptomycin at 37 °C in a 5% CO_2_ incubator. The cells were passaged two to three times a day. Following in vitro cultivation, trypsinized cells were harvested, rinsed, and counted using the trypan blue exclusion assay. Subsequently, the cells were centrifuged, re-dispersed in phosphate-buffered saline (PBS; pH 7.2), and adjusted to a concentration of 1.0 × 10^7^ cells/mL before transplantation.

### 2.3. Animal Experimental Protocol

Forty female BALB/c mice were obtained from Yisi Laboratory Animal Technology Co., Limited (Changchun, China), and the mice were subsequently maintained at 23–25 °C with 50–55% humidity under a 12 h light/dark cycle. Following a 1-week acclimatization period, the animals were provided with standard chow and drinking water. A dose of 1.0 × 10^7^ viable tumor cells was suspended in 0.2 mL PBS and injected subcutaneously into the right scapula of the mice. After a tumor with an average diameter of approximately 5 mm was visible at the inoculation site, the animals were randomly distributed into four experimental groups, with each group comprising ten individuals. In the model group, cyclophosphamide group (CY), probiotic group (ProB), and postbiotic group (PostB), gavage was then initiated. *W. coagulans* MZY531 (1.0 × 10^9^ CFU/mL, 10 mL/kg/d) and *W. coagulans* MZY531 postbiotics (10 mL/kg/d) were administered to the ProB and PostB groups. The model group was gavaged with saline (10 mL/kg/d), and the CY group was injected intraperitoneally with cyclophosphamide at 30 mg/kg/d.

Body weight, along with tumor length (a) and width (b), were recorded every 3 d. The tumor volume was subsequently calculated based on these measurements as V (cm^3^) = (a × b^2^) × 1/2, and the tumor growth curve was plotted. After administering the final dose on day 21, upon euthanasia, serum and tumor tissues were retrieved, measured, and preserved at −80 °C for subsequent analysis. The tumor inhibition rate was determined using the following formula [22]:

Tumor inhibition rate = 100% × (mean tumor weight of the control group—mean tumor weight of the treatment group)/mean tumor weight of the control group.

### 2.4. Serum Biochemistry Assay

The levels of mouse colon cancer antigen (CCA), carcinoembryonic antigen (CEA), and extracellular protein kinase A (ECPKA) in serum were automatically detected using ELISA kits. The ELISA kits were provided by Jiangsu Jingmei Biotechnology Co., Ltd. (Yancheng, China).

### 2.5. Immunofluorescence Staining Assays

Tumor tissues were fixed in 10% formalin (Tianjin, China) overnight before being embedded in paraffin (Wuhan, China). Sections were cut into slices 10 μm thick, incubated with PBS containing bovine serum albumin (3%) and Triton X-100 (0.3%) for 1 h, and then treated with TH antibody (Servicebio, GB11532, Wuhan, China) incubated at 4 °C for 12 h. Next, incubation with a secondary antibody (Servicebio, GB21301, Wuhan, China) at room temperature was carried out for 1 h, along with subsequent incubation with diaminobenzidine for approximately 10 min. The expression of caspase-3 was observed under an optical microscope, and the positive cell rate of caspase-3 was calculated using ImageJ v1.8.0 software (National Institutes of Health, Bethesda, MD, USA).

### 2.6. In Vivo Apoptosis Induction by W. coagulans MZY531

Paraffin sections of the tumor were first dewaxed using xylene and then subjected to a gradient ethanol dehydration process, followed by washing and drying in PBS. After incubation with proteinase K at room temperature for 20 min, the samples were rinsed with PBS. The terminal deoxynucleotidyl transferase dUTP nick-end labeling (TUNEL) working solution was added to the sections and then covered with a thermal film and incubated at 37 °C for 2 h in the dark. The samples were washed three times with PBS, dried, and incubated with DAPI solution to stain the nuclei. Apoptotic cells were observed under a fluorescence microscope, and images were captured.

### 2.7. Western Blotting

Tumor tissue was lysed using RIPA buffer and then centrifuged at 10,000× *g* for 10 min at 4 °C to remove insoluble debris. The protein concentration was quantified using a BCA protein assay kit (UElandy, Suzhou, China). Protein samples were resolved using sodium dodecyl sulfate–polyacrylamide gel electrophoresis and subsequently transferred onto polyvinylidene fluoride membranes (Millipore, Billerica, MA, USA). Following blocking with 5% bovine serum albumin, the membranes were incubated with the appropriate antibodies: β-actin, p-JAK2, STAT3, Bcl-2 and Bax (GeneTex, San Antonio, TX, USA), JAK2, p-STAT3, PI3K, AKT, mTOR and p-mTOR (Bioss, Beijing, China), caspase-3 (Abcam, Cambridge, MA, USA), TGF-β, and SMAD4 (Proteintech, Wuhan, China). After three washes with TBST (BestEnzymes, Lianyugang, China), membranes were incubated with horseradish peroxi-dase-conjugated antibodies. Protein levels were detected using a chemiluminescence assay kit (Millipore, MA, USA). The density analysis of immunoblots was conducted using Image v1.8.0 software (National Institutes of Health, Bethesda, MD, USA).

### 2.8. Statistical Analysis

Statistical analysis was conducted using a one-way ANOVA in SPSS Statistics 23 (IBM Corp., Armonk, NY, USA). Significant group differences (*p* < 0.05) were assessed using Duncan’s multiple range test, where means are ranked as a > b > c > d. Data are presented as mean ± standard deviation.

## 3. Results

### 3.1. W. coagulans MZY531 Postbiotics Ameliorated Tumor Growth

**Figure 1:**
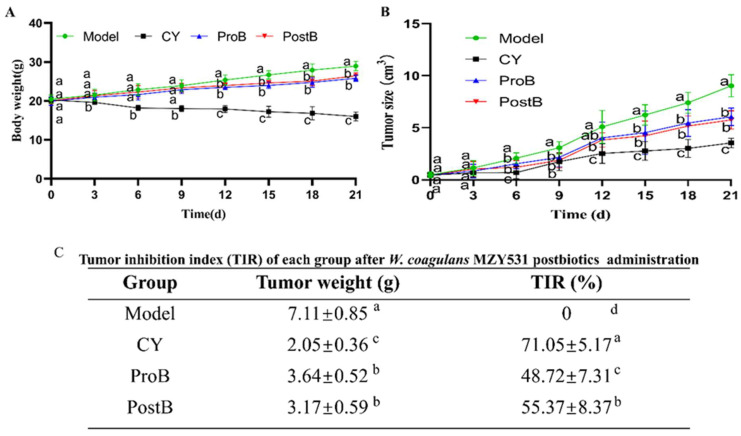
Inhibitory effects of *W. coagulans* MZY531 postbiotics on CT26.WT colorectal cancer growth in mice. (**A**) The change in body weight during the experimental period, (**B**) the size of the tumor, and (**C**) the tumor inhibition index. Statistical analysis was conducted using ANOVA, with subsequent post hoc testing performed via Duncan’s multiple comparison test. Means with different superscript letters (a, b, c, and d) are statistically significantly different from each other (*p* < 0.05), with ‘a’ representing the highest value and ‘d’ representing the lowest.

Changes in the body weight and tumor size of mice during the experiment are shown in Figure 1. The ProB and PostB groups exhibited lower body weights compared with those of the model group, with statistically significant differences observed between these groups and the model group (Figure 1A). The tumor volume in the model group increased rapidly. Compared with those in the model group, tumor volumes were significantly reduced in the ProB and PostB groups, with the PostB group exhibiting smaller tumor sizes than those of the ProB group (Figure 1B). The results of the tumor weight and tumor inhibition index calculations are illustrated in Figure 1C. The tumor weight in the model group was 7.11 ± 0.85 g. Compared to the model group, the tumor weights in the CY, ProB, and PostB groups were significantly reduced, measuring 2.05 ± 0.36 g, 3.64 ± 0.52 g, and 3.17 ± 0.59 g, respectively. The tumor inhibition rates were 71.05 ± 5.17% for the CY group, 48.72 ± 7.31% for the ProB group, and 55.37 ± 8.37% for the PostB group. Notably, both ProB and PostB treatments significantly enhanced the tumor inhibition index compared to the model group, with the PostB group exhibiting a more pronounced effect than the ProB group.

### 3.2. W. coagulans MZY531 Postbiotics Regulate Tumor Markers’ Cytokines in Serum

**Figure 2:**
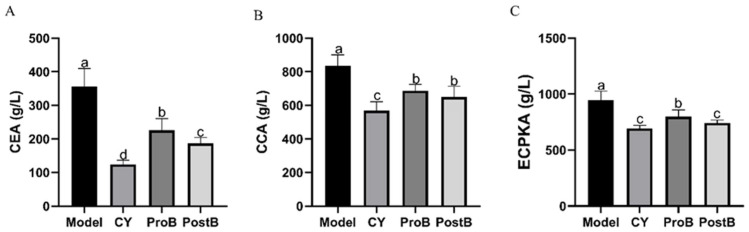
Effect of *W. coagulans* MZY531 postbiotics on improving serum tumor markers parameters of mice. (**A**) Impact of postbiotics on CEA levels of serum, (**B**) impact of postbiotics on CCA levels of serum, and (**C**) impact of postbiotics on ECPKA levels of serum. Statistical analysis was conducted using ANOVA, with subsequent post hoc testing performed via Duncan’s multiple comparison test. Means with different superscript letters (a, b, c, and d) are statistically significantly different from each other (*p* < 0.05), with ‘a’ representing the highest value and ‘d’ representing the lowest.

Postbiotics that regulate the tumor markers CEA, CCA, and ECPKA showed significant differences (Figure 2). CEA expression levels in the ProB and PostB groups were significantly lower than those in the model group, with the levels of the PostB group showing a significantly greater reduction than those of the ProB group (Figure 2A). Compared with those in the model group, CCA levels in the ProB and PostB groups were significantly reduced (Figure 2B). ECPKA levels in the ProB and PostB groups were significantly lower than those in the model group. Furthermore, the PostB group exhibited significantly greater reductions than those of the ProB group, with no significant difference observed when compared with the reductions in the CY group (Figure 2C).

### 3.3. W. coagulans MZY531 Postbiotics Regulate the Expression of Caspase-3 in Tumor Tissues

**Figure 3:**
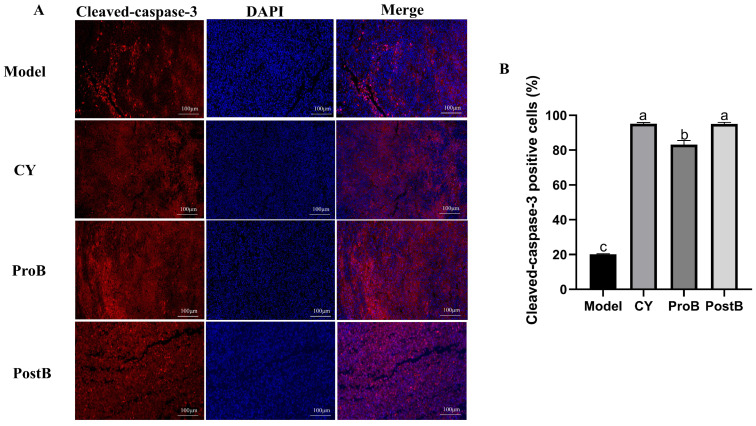
Effect of *W. coagulans* MZY531 postbiotics on caspase-3 expression in the tumor tissue of mice. (**A**) Immunofluorescence staining was performed at 20× magnification for cleaved-caspase-3-positive cells in mice tumor tissue, and (**B**) the proportion of cleaved-caspase-3-positive cells in each group. Statistical analysis was conducted using ANOVA, with subsequent post hoc testing performed via Duncan’s multiple comparison test. Means with different superscript letters (a, b, and c) are statistically significantly different from each other (*p* < 0.05), with ‘a’ representing the highest value and ‘c’ representing the lowest.

Figure 3A,B show the proportion of cleaved-caspase-3-positive cells in mouse tumor tissues. The ProB and PostB groups exhibited significantly higher counts of cleaved-caspase-3-positive cells in the tumor tissues than those of the model group. There was no significant difference in the cleaved-caspase-3 positive cell rate between the PostB and CY groups.

### 3.4. W. coagulans MZY531 Postbiotics Modulate TUNEL Expression in Tumor Tissues

**Figure 4:**
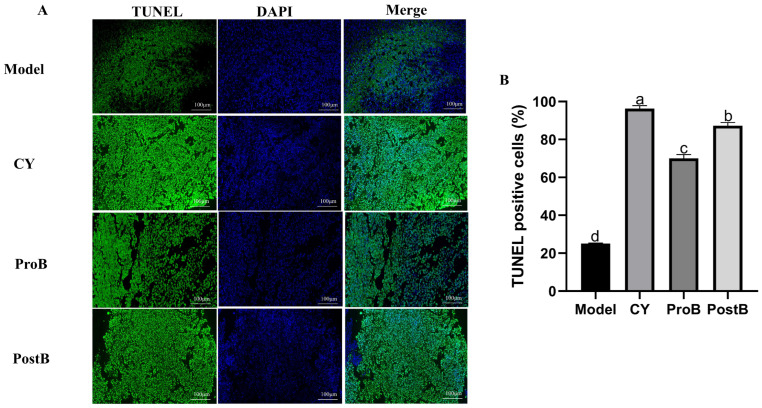
Effect of *W. coagulans* MZY531 postbiotics on TUNEL expression in tbe tumor tissue of mice. (**A**) TUNEL-positive cells in mouse tumor tissue were visualized using immunofluorescence staining with 20× magnification. (**B**) The positive rate of TUNEL-positive cells in each group. Statistical analysis was conducted using ANOVA, with subsequent post hoc testing performed via Duncan’s multiple comparison test. Means with different superscript letters (a, b, c, and d) are statistically significantly different from each other (*p* < 0.05), with ‘a’ representing the highest value and ‘d’ representing the lowest.

The TUNEL-positive cell assay results are shown in Figure 4A,B. The number of positive cells was significantly higher in the PostB group than in the ProB group. Additionally, the positive cell rates in both the ProB and PostB groups were significantly higher than those in the model group.

### 3.5. W. coagulans MZY531 Postbiotics Promote Tumor Apoptosis Through the Modulation of the Bax/Bcl-2/Caspase-3 and JAK2/STAT3 Signaling Pathways

**Figure 5:**
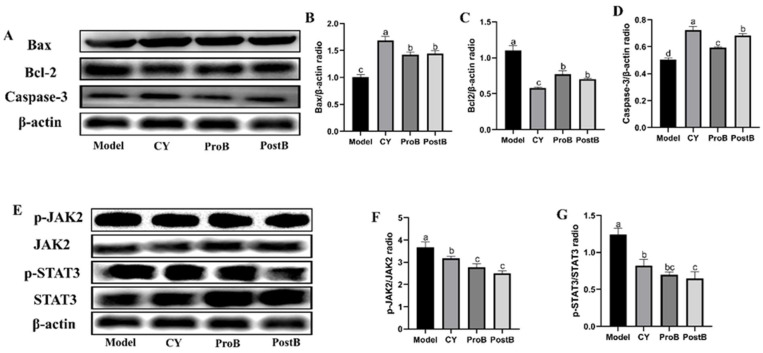
Western blotting diagram of the effect of *W. coagulans* MZY531 postbiotics on the expression of apoptosis signaling pathway proteins. (**A**) Bax, Bcl2, and caspase-3 protein expressions in tumor tissue. (**B**–**D**) Bax, Bcl2, and caspase-3 protein expression gray value. (**E**) Phosphorylation of JAK2 and STAT3 and the expression levels of unphosphorylated JAK2 and STAT3 proteins in tumor tissue. (**F**,**G**) P-JAK2/JAK2 and P-STAT3/STAT3 protein expression gray value. Statistical analysis was conducted using ANOVA, with subsequent post hoc testing performed via Duncan’s multiple comparison test. Means with different superscript letters (a, b, c, and d) are statistically significantly different from each other (*p* < 0.05), with ‘a’ representing the highest value and ‘d’ representing the lowest.

We investigated whether the Bax/Bcl2/caspase-3 and JAK2/STAT3 pathways were involved in promoting the apoptotic mechanism of *W. coagulans* MZY531 postbiotics in mice (Figure 5). Compared with those of the model group, the ProB and PostB groups showed significantly reduced protein expression levels of Bcl-2 (Figure 5C). By contrast, Bax and caspase-3 levels dramatically increased in the ProB and PostB groups compared with those in the model group (Figure 5B,D). As shown in Figure 5F,G, phosphorylated JAK2 and STAT3 decreased significantly in the ProB and PostB groups, whereas the expression of JAK and STAT3 increased significantly. Therefore, the ProB and PostB groups showed significant therapeutic effects compared with those of the model group.

### 3.6. W. coagulans MZY531 Postbiotics Promote the Autophagy of Tumors by the PI3K/AKT/mTOR and TGF-β/SMAD4 Signaling Pathways

**Figure 6:**
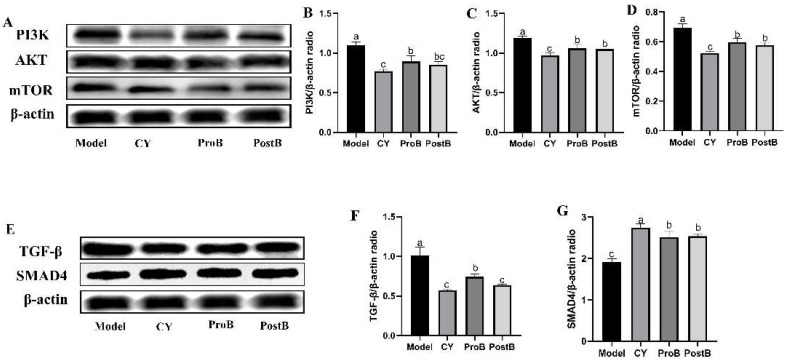
Western blotting diagram analysis illustrating the impact of W. coagulans MZY531 postbiotics on the expression of proteins involved in the autophagy signaling pathway. (**A**) PI3K, AKT, and mTOR protein expressions in tumor tissue. (**B**–**D**) PI3K, AKT, and mTOR protein expression gray value. (**E**) TGF-β and SMAD 4 protein expressions in tumor tissue. (**F**,**G**) TGF-β and SMAD 4 protein expression gray value. Statistical analysis was conducted using ANOVA, with subsequent post hoc testing performed via Duncan’s multiple comparison test. Means with different superscript letters (a, b, and c) are statistically significantly different from each other (*p* < 0.05), with ‘a’ representing the highest value and ‘c’ representing the lowest.

To elucidate the mechanism underlying the inhibition of tumor growth mediated by PostB, we examined the protein expression of autophagy pathway markers, such as PI3K, AKT, mTOR, TGF-β, and SMAD4, in the tumor tissues of CT26.WT-bearing mice by Western blotting (Figure 6). Compared with those of the model group, the ProB and PostB groups showed significantly reduced protein expressions of PI3K, AKT, and mTOR (Figure 6B–D). Compared with those of the model group, the ProB and PostB groups showed significantly reduced protein expression levels of TGF-β. Differences between the CY and PostB groups were not statistically significant. Treatment with postbiotics significantly increased SMAD4 in the ProB and PostB groups. This mechanism may be critical in explaining the pharmacological properties of *W. coagulans* MZY531.

## 4. Discussion

CRC tumor markers are important indicators for evaluating the occurrence of CRC and monitoring disease recurrence in patients after treatment. The detection of elevated CEA levels may raise suspicion of the underlying development of CRC and recurrence after treatment for colon or rectal cancer [23]. CEA is a promising targeted biomarker for the clinical monitoring of patients with CRC [24]. This study confirmed that higher CEA values may indicate advanced CRC and worse outcomes [25]. In the present study, postbiotic supplementation significantly reduced the CEA levels in serum. Chang et al. showed that *Butyricicoccus pullicaecorum*-supplemented CRC-bearing mice exhibited significantly reduced serum CEA levels [26]. ECPKA is significantly elevated in the serum of patients with cancer and is a potential diagnostic marker of cancer [27,28]. *W. coagulans* MZY531 postbiotics supplementation significantly improved ECPKA levels in serum. In addition, our intriguing discovery was that intragastric delivery of *W. coagulans* MZY531 postbiotics markedly reduced CCA expression in serum. Therefore, the decrease in CEA, CCA, and ECPKA levels in serum demonstrated that *W. coagulans* MZY531 postbiotics may be efficacious in CRC therapy.

Cell-induced apoptosis is associated with cancer treatment [29]. TUNEL immunofluorescence staining is an important method for measuring tumor cell apoptosis [30]. Zhou et al. evaluated the effect of *Clostridium butyricum* supplementation on cancer cell apoptosis using the TUNEL staining test [31]. Our TUNEL staining results were consistent with those of previous studies. Supplementation with *W. coagulans* MZY531 postbiotics significantly increased the percentage of TUNEL-positive cells. Caspase-3 enzyme activity in tumor cells gradually increased in a concentration-dependent manner, and an increase in caspase-3 immunofluorescence indicated the occurrence of cell apoptosis [32]. An increase in caspase-3-positive cells is an important mechanism for effectively improving CRC [33]. Our study demonstrates that the rate of caspase-3-positive cells increased significantly after postbiotic supplementation. A growing body of evidence indicates that the Bax/Bcl-2 and caspase-3 pathway is implicated in the amelioration of CRC [34]. Additionally, the JAK family of proteins plays a role in apoptosis. JAK2 activation occurs through phosphorylation, which facilitates the phosphorylation of downstream molecules, such as STAT3. Moreover, the JAK2/STAT3 pathway exhibits significant anticancer activity [33,35]. Therefore, we analyzed the Bax/Bcl-2/caspase-3 and JAK2/STAT3 pathways to elucidate the mechanism by which probiotics improve colorectal progression. Our study demonstrated that the apoptotic factors Bax, Bcl-2, caspase-3, P-JAK2, JAK2, P-STAT3, and STAT3 were significantly upregulated and improved after postbiotic supplementation. This result is consistent with the previous results of Nafiseh Erfanian [36], Morteza Banakar [37], and Suki Ha [38], who found that postbiotics regulate tumors by improving cell apoptosis signaling pathways. *W. coagulans* MZY531 postbiotics modulate cell apoptosis and may represent a crucial mechanism for enhancing CRC treatment.

The regulation of autophagy is a promising strategy for cancer treatment [39]. The hyperactivation of PI3K and gain-of-function mutations in AKT are well-known contributors to disease progression and resistance to cancer [40]. TGF-β overexpression is significantly related to tumorigenesis. SMAD4 functions as a key regulator within the TGF-β signaling pathway, and multiple studies have shown that it does not independently initiate tumor formation. However, the loss of SMAD4 can facilitate tumor progression initiated by other oncogenic factors [41,42]. The results of this study showed that in the model mice, there was an increase in the expression of PI3K/AKT/mTOR and TGF-β proteins and a reduction in SMAD4. However, treatment with *W. coagulans* MZY531 inhibited the activation of PI3K/AKT/mTOR pathways. Our results further confirm the findings of Zhao et al., who found that the modulation of the PI3K, AKT, and mTOR signaling pathways by *W. coagulans* MZY531 is an important mechanism for improving cancer [21].

Simultaneously, studies have shown that probiotics can enhance anti-CRC effects by blocking TGF-β expression. Our research proves that TGF-β inhibition mediated the activation of SMAD4, leading to the activation of cellular autophagy. Therefore, *W. coagulans* MZY531 postbiotics may improve CRC by enhancing autophagy.

## 5. Conclusions

Our findings suggest that *W. coagulans* MZY531 postbiotics have positive effects on CRC. *W. coagulans* MZY531 postbiotics improved the levels of tumor marker cytokines and elevated the quantity of caspase-3- and TUNEL-positive cells, concomitant with a reduction in tumor growth. In addition, *W. coagulans* MZY531 regulates the Bax/Bcl-2/caspase-3 and JAK2/STAT3 apoptosis pathways and PI3K/AKT/mTOR pathways, as well as TGF-β/SMAD4 autophagy pathways, thereby attenuating tumor growth. These results suggest a novel therapeutic strategy for CRC treatment and prevention.

## Data Availability

The data presented in this study are available upon request from the corresponding author.

## References

[1] Xi Y., Xu P. (2021). Global colorectal cancer burden in 2020 and projections to 2040. Transl. Oncol..

[2] Morgan E., Arnold M., Gini A., Lorenzoni V., Cabasag C.J., Laversanne M., Vignat J., Ferlay J., Murphy N., Bray F. (2023). Global burden of colorectal cancer in 2020 and 2040: Incidence and mortality estimates from GLOBOCAN. Gut.

[3] Keum N., Giovannucci E. (2019). Global burden of colorectal cancer: Emerging trends, risk factors and prevention strategies. Nat. Rev. Gastroenterol. Hepatol..

[4] Piawah S., Venook A.P. (2019). Targeted therapy for colorectal cancer metastases: A review of current methods of molecularly targeted therapy and the use of tumor biomarkers in the treatment of metastatic colorectal cancer. Cancer.

[5] Zhang C., Stampfl-Mattersberger M., Ruckser R., Sebesta C. (2023). [Colorectal cancer]. Wien Med. Wochenschr..

[6] Islam M.R., Akash S., Rahman M.M., Nowrin F.T., Akter T., Shohag S., Rauf A., Aljohani A.S.M., Simal-Gandara J. (2022). Colon cancer and colorectal cancer: Prevention and treatment by potential natural products. Chem. Biol. Interact..

[7] Gou H., Su H., Liu D., Wong C.C., Shang H., Fang Y., Zeng X., Chen H., Li Y., Huang Z. (2023). Traditional Medicine Pien Tze Huang Suppresses Colorectal Tumorigenesis Through Restoring Gut Microbiota and Metabolites. Gastroenterology.

[8] Torres-Maravilla E., Boucard A.S., Mohseni A.H., Taghinezhad S.S., Cortes-Perez N.G., Bermudez-Humaran L.G. (2021). Role of Gut Microbiota and Probiotics in Colorectal Cancer: Onset and Progression. Microorganisms.

[9] Eslami M., Yousefi B., Kokhaei P., Hemati M., Nejad Z.R., Arabkari V., Namdar A. (2019). Importance of probiotics in the prevention and treatment of colorectal cancer. J. Cell. Physiol..

[10] de Moreno de LeBlanc A., Matar C., Perdigon G. (2007). The application of probiotics in cancer. Br. J. Nutr..

[11] Molska M., Regula J. (2019). Potential Mechanisms of Probiotics Action in the Prevention and Treatment of Colorectal Cancer. Nutrients.

[12] Zhao J., Liao Y., Wei C., Ma Y., Wang F., Chen Y., Zhao B., Ji H., Wang D., Tang D. (2023). Potential Ability of Probiotics in the Prevention and Treatment of Colorectal Cancer. Clin. Med. Insights Oncol..

[13] Vinderola G., Sanders M.E., Salminen S. (2022). The Concept of Postbiotics. Foods.

[14] Zolkiewicz J., Marzec A., Ruszczynski M., Feleszko W. (2020). Postbiotics-A Step Beyond Pre- and Probiotics. Nutrients.

[15] Kvakova M., Kamlarova A., Stofilova J., Benetinova V., Bertkova I. (2022). Probiotics and postbiotics in colorectal cancer: Prevention and complementary therapy. World J. Gastroenterol..

[16] Mu Y., Cong Y. (2019). Bacillus coagulans and its applications in medicine. Benef. Microbes.

[17] Konuray G., Erginkaya Z. (2018). Potential Use of Bacillus coagulans in the Food Industry. Foods.

[18] Sasaki K., Sasaki D., Inoue J., Hoshi N., Maeda T., Yamada R., Kondo A. (2020). Bacillus coagulans SANK 70258 suppresses Enterobacteriaceae in the microbiota of ulcerative colitis in vitro and enhances butyrogenesis in healthy microbiota. Appl Microbiol. Biotechnol..

[19] Madempudi R.S., Kalle A.M. (2017). Antiproliferative Effects of Bacillus coagulans Unique IS2 in Colon Cancer Cells. Nutr. Cancer.

[20] Dolati M., Tafvizi F., Salehipour M., Movahed T.K., Jafari P. (2021). Inhibitory effects of probiotic Bacillus coagulans against MCF7 breast cancer cells. Iran. J. Microbiol..

[21] Zhao Z., Yang Q., Zhou T., Liu C., Sun M., Cui X., Zhang X. (2023). Anticancer potential of Bacillus coagulans MZY531 on mouse H22 hepatocellular carcinoma cells via anti-proliferation and apoptosis induction. BMC Complement. Med. Ther..

[22] Lu X.G., Zhan L.B., Feng B.A., Qu M.Y., Yu L.H., Xie J.H. (2004). Inhibition of growth and metastasis of human gastric cancer implanted in nude mice by d-limonene. World J. Gastroenterol..

[23] Woolfson K. (1991). Tumor markers in cancer of the colon and rectum. Dis. Colon Rectum.

[24] Campos-da-Paz M., Dorea J.G., Galdino A.S., Lacava Z.G.M., de Fatima Menezes Almeida Santos M. (2018). Carcinoembryonic Antigen (CEA) and Hepatic Metastasis in Colorectal Cancer: Update on Biomarker for Clinical and Biotechnological Approaches. Recent Pat. Biotechnol..

[25] Lerch M., Sengul D., Sengul I., Peteja M., Pribylova L., Gawel W., Pelikan A., Tomaskova H., Maly T., Janout V. (2023). Revisiting ab initio carcinoembryonic antigen and CA19-9 tumor markers in colorectal carcinoma in association with anatomotopographic location and staging of disease. Rev. Assoc. Med. Bras. (1992).

[26] Chang S.C., Shen M.H., Liu C.Y., Pu C.M., Hu J.M., Huang C.J. (2020). A gut butyrate-producing bacterium Butyricicoccus pullicaecorum regulates short-chain fatty acid transporter and receptor to reduce the progression of 1,2-dimethylhydrazine-associated colorectal cancer. Oncol. Lett..

[27] Cho Y.S., Park Y.G., Lee Y.N., Kim M.K., Bates S., Tan L., Cho-Chung Y.S. (2000). Extracellular protein kinase A as a cancer biomarker: Its expression by tumor cells and reversal by a myristate-lacking Calpha and RIIbeta subunit overexpression. Proc. Natl. Acad. Sci. USA.

[28] Nesterova M.V., Johnson N., Cheadle C., Bates S.E., Mani S., Stratakis C.A., Khan I.U., Gupta R.K., Cho-Chung Y.S. (2006). Autoantibody cancer biomarker: Extracellular protein kinase A. Cancer Res..

[29] Tvrdik D., Skalova H., Dundr P., Povysil C., Velenska Z., Berkova A., Stanek L., Petruzelka L. (2012). Apoptosis—Associated genes and their role in predicting responses to neoadjuvant breast cancer treatment. Med. Sci. Monit..

[30] Shen J., Li Z.J., Li L.F., Lu L., Xiao Z.G., Wu W.K., Zhang L., Li M.X., Hu W., Chan K.M. (2016). Vascular-targeted TNFalpha and IFNgamma inhibits orthotopic colorectal tumor growth. J. Transl. Med..

[31] Zhou M., Yuan W., Yang B., Pei W., Ma J., Feng Q. (2022). Clostridium butyricum inhibits the progression of colorectal cancer and alleviates intestinal inflammation via the myeloid differentiation factor 88 (MyD88)-nuclear factor-kappa B (NF-kappaB) signaling pathway. Ann. Transl. Med..

[32] Pandurangan M., Mistry B., Enkhataivan G., Kim D.H. (2016). Efficacy of carnosine on activation of caspase 3 and human renal carcinoma cell inhibition. Int. J. Biol. Macromol..

[33] Shen Y., Gao Y., Yang G., Zhao Z., Zhao Y., Gao L., Zhao L., Li S. (2023). Transformation of Ginsenosides by Lactiplantibacillus plantarum MB11 Fermentation: Minor Ginsenosides Conversion and Enhancement of Anti-Colorectal Cancer Activity. Molecules.

[34] Huang S., Cui M., Wang R., Yang G., Wang N., Cui L., Ma G. (2023). Combined treatment with Prunella vulgaris and Radix bupleuri activated the Bax/Bcl-2 and Caspase-3 signal pathways in papillary thyroid carcinoma cells. Nucleosides Nucleotides Nucleic Acids.

[35] Park S.Y., Lee C.J., Choi J.H., Kim J.H., Kim J.W., Kim J.Y., Nam J.S. (2019). The JAK2/STAT3/CCND2 Axis promotes colorectal Cancer stem cell persistence and radioresistance. J. Exp. Clin. Cancer Res..

[36] Erfanian N., Safarpour H., Tavakoli T., Mahdiabadi M.A., Nasseri S., Namaei M.H. (2024). Investigating the therapeutic potential of Bifidobacterium breve and Lactobacillus rhamnosus postbiotics through apoptosis induction in colorectal HT-29 cancer cells. Iran. J. Microbiol..

[37] Banakar M., Etemad-Moghadam S., Haghgoo R., Mehran M., Yazdi M.H., Mohamadpour H., Iravani Saadi M., Alaeddini M. (2023). Anticancer Activity of Postbiotic Mediators Derived from Lactobacillus Rhamnosus GG and Lactobacillus Reuteri on Acute Lymphoblastic Leukemia Cells. Galen Med. J..

[38] Ha S., Zhang X., Yu J. (2024). Probiotics intervention in colorectal cancer: From traditional approaches to novel strategies. Chin. Med. J..

[39] Li X., He S., Ma B. (2020). Autophagy and autophagy-related proteins in cancer. Mol. Cancer.

[40] Glaviano A., Foo A.S.C., Lam H.Y., Yap K.C.H., Jacot W., Jones R.H., Eng H., Nair M.G., Makvandi P., Geoerger B. (2023). PI3K/AKT/mTOR signaling transduction pathway and targeted therapies in cancer. Mol. Cancer.

[41] Zhao M., Mishra L., Deng C.X. (2018). The role of TGF-beta/SMAD4 signaling in cancer. Int. J. Biol. Sci..

[42] Yang T., Huang T., Zhang D., Wang M., Wu B., Shang Y., Sattar S., Ding L., Liu Y., Jiang H. (2019). TGF-beta receptor inhibitor LY2109761 enhances the radiosensitivity of gastric cancer by inactivating the TGF-beta/SMAD4 signaling pathway. Aging.

